# *Solanum anguivi* Lam. Fruits: Their Potential Effects on Type 2 Diabetes Mellitus

**DOI:** 10.3390/molecules26072044

**Published:** 2021-04-02

**Authors:** Aisha Musaazi Sebunya Nakitto, John H. Muyonga, Yusuf Byenkya Byaruhanga, Anika E. Wagner

**Affiliations:** 1 Department of Food Technology and Nutrition, School of Food Technology Nutrition and Bioengineering, College of Agricultural and Environmental Sciences, Makerere University, P.O. Box 7062 Kampala, Uganda or aishasebunya@gmail.com (A.M.S.N.); hmuyonga@yahoo.com (J.H.M.); ybbyaru@gmail.com (Y.B.B.); 2 Institute of Nutritional Sciences, Justus-Liebig University, Wilhelmstrasse 20, 35392 Giessen, Germany

**Keywords:** type 2 diabetes, *Solanum anguivi* fruits, antioxidants, pathogenesis of diabetes, bioactivity, oxidative stress, antidiabetic, glucose homeostasis

## Abstract

Type 2 diabetes mellitus (T2DM) is a complex metabolic disorder of glucose homeostasis associated with a status of insulin resistance, impaired insulin signaling, β-cell dysfunction, impaired glucose and lipid metabolism, sub-clinical inflammation, and increased oxidative stress. Consuming fruits and vegetables rich in phytochemicals with potential antidiabetic effects may prevent T2DM and/or support a conservative T2DM treatment while being safer and more affordable for people from low-income countries. *Solanum anguivi* Lam. fruits (SALF) have been suggested to exhibit antidiabetic properties, potentially due to the presence of various phytochemicals, including saponins, phenolics, alkaloids, ascorbic acid, and flavonoids. For the saponin fraction, antidiabetic effects have already been reported. However, it remains unclear whether this is also true for the other phytochemicals present in SALF. This review article covers information on glucose homeostasis, T2DM pathogenesis, and also the potential antidiabetic effects of phytochemicals present in SALF, including their potential mechanisms of action.

## 1. Introduction

Diabetes is a chronic metabolic disorder that is illustrated by either insufficient production or the lack of response to insulin, a key hormone in the regulation of the body’s metabolism [1]. The burden, due to diabetes is enormous, owing to its rapidly increasing global prevalence, the devastating damage it can do to many body organs, and the direct and indirect costs [2]. The estimated global prevalence of diabetes in people aged 20–79 years has risen from 6.4% (285 million) in 2010 to 9.3% (463 million) in 2019, and it is predicted to increase to 10.9% (700 million) by 2045 if there is insufficient action to address the pandemic [3]. Based on the World Bank income classification, high-income countries had the highest diabetes prevalence in 2019 at 10% (95.2 million), while low-income countries had the least at 4% (14.5 million) [3]. 

People suffer from different types of diabetes, including type 1 diabetes mellitus (T1DM), type 2 diabetes mellitus (T2DM), gestational diabetes mellitus, monogenic diabetes, and secondary diabetes [4]. T2DM is the most common type of diabetes [4], and will, therefore, be the focus of this review article. The present review provides information on glucose homeostasis and how T2DM ensues (pathogenesis of T2DM). A few studies [5,6] suggest antidiabetic properties of *Solanum anguivi* Lam. Fruits (SALF), due to the presence of bioactive phytochemical compounds. *Solanum anguivi* Lam. is an ethnomedicinal plant belonging to the family Solanaceae and genus *Solanum* Lam [7]. It is native to Africa, probably occurring in all non-arid tropical African regions [8], and it has also been reported to be present in Asia and Australia [9]. It grows mostly in the wild, but sometimes, e.g., in Uganda and Ivory Coast, it is a semi-cultivated vegetable [8]. The plants are consumed as leafy and/or fruity vegetables [10]. However, only limited data regarding its antidiabetic effect is available, which will be summarized in this review, as well as the potential mechanisms of action for phytochemicals present in SALF that may lead to antidiabetic effects. 

## 2. Glucose Homeostasis

**Figure 1:**
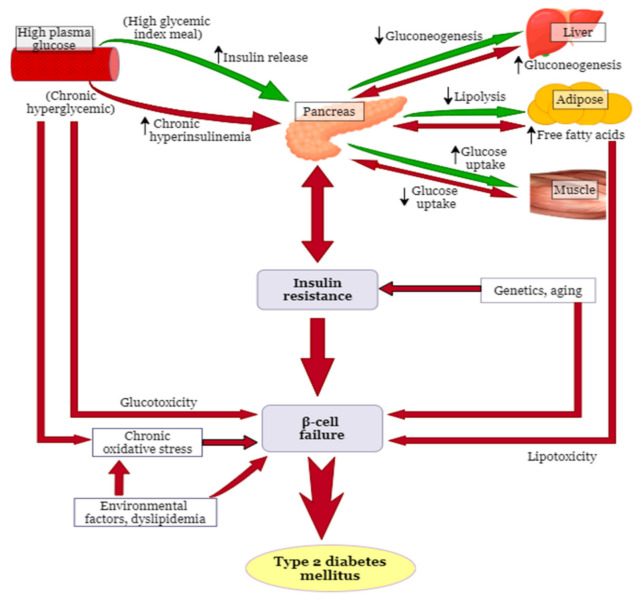
Normal glucose homeostasis and the pathogenesis of type 2 diabetes mellitus. This was modified, according to Ludwig [16]. The green arrows show normal glucose homeostasis, while the red arrows show the pathogenesis of type 2 diabetes mellitus. The black upward arrows represent an increase, while the downward ones represent inhibition. High plasma glucose may result from a high glycemic meal, or it may be during chronic hyperglycemia, leading to increased insulin production or chronic hyperinsulinemia, respectively. The events that follow are shown by the green and red arrows, respectively. The figure was drawn via https://app.diagrams.net/, and the pancreas, liver, and muscle pictures were obtained from www.freepik.com.

The pancreas maintains blood glucose levels within a very narrow range of 4.0–6.5 mmol/L [11] mediated through the opposing and balanced actions of the hormones glucagon and insulin, referred to as glucose homeostasis [12]. Glucagon and insulin are synthesized from the pancreatic α- and β- cells of the islets of Langerhans, respectively [13]. The systemic glucose homeostasis is achieved by the coordinated functions of different organ systems, including the skeletal muscle, the liver, the endocrine pancreas, the adipose tissue (Figure 1) [14], and the hypothalamus is responsible for the neural regulation of these organ systems [15].

### Insulin and Glucagon as Mediators of the Glucose Homeostasis

**Figure 2:**
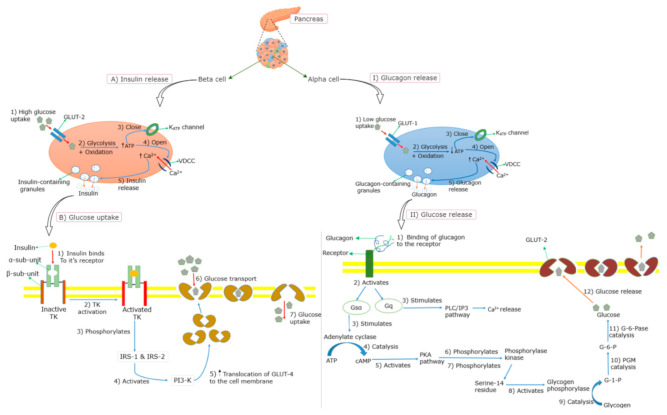
Insulin and glucagon secretion mechanisms. (**A**) = insulin release during high glucose concentration, (**B**) = insulin-dependent glucose uptake, (**I**) = glucagon release during low glucose concentration, (**II**) conversion of glucagon to glucose. GLUT = glucose transporter, VDDC = voltage-dependent calcium channel, ATP = adenosine triphosphate, TK= tyrosine kinase, IRS = insulin receptor substrate, PI3-K = phosphatidylinositol-3-kinase, cAMP = cyclic adenosine monophosphate, PKA = protein kinase A, G-1-P = glucose-1-phosphate, PGM = Phosphoglucomutase, G-6-P = glucose-6-phosphate, G-6-Pase = glucose-6-phosphatase, PLC = phospholipase C and IP3 = inositol 1,4,5-triphosphate. The events are shown by numbered steps using arrows: Red = entry, blue = resulting to, orange = exocytosis; while black upward arrow = increased content. The green arrows were used for labelling.

The main stimulus for the insulin release from the pancreatic β-cells is an elevated blood glucose level following the ingestion of glucose or a high-glycemic-index meal (≥65 on the glucose scale [17]) [18]. The circulating plasma glucose is taken up into the β-cells through the facilitative glucose transporter (GLUT) in an insulin-independent manner [19,20] (Figure 2). Once in the β-cell, glucose undergoes glycolysis and mitochondrial glucose oxidation, leading to an increased adenosine triphosphate (ATP)/adenosine diphosphate (ADP) ratio and the subsequent closure of ATP-sensitive potassium (K+) channels (K_ATP_ channels). This leads to the depolarization of the membrane, followed by the opening of voltage-dependent calcium (Ca^2+^) channels (VDCCs), resulting in the influx of Ca^2+^ and the eventual release of insulin [12,19,21,22]. Insulin binds to the α-subunit of the insulin receptor, which enables ATP to bind to the β-subunit of the insulin receptor, which in turn triggers the phosphorylation of the tyrosine kinase [23,24] (Figure 2). Several intracellular proteins are then phosphorylated on tyrosine residues, such as insulin receptor substrates (IRS) 1 and 2, leading to the activation of phosphatidylinositol-3-kinase (PI3-K) [25,26]. This subsequently increases the translocation of GLUT-4 molecules on the outer membrane of the insulin-responsive tissues [23,27], leading to an increased glucose uptake. Insulin-mediated signaling further lowers blood glucose by reducing hepatic glucose output (gluconeogenesis) by increasing the storage of glucose as glycogen in the liver (glycogenesis) and inhibiting the release of free fatty acids (FFAs) from adipose tissue (lipolysis) through promoting fat synthesis (lipogenesis) in the adipose tissue [28,29]. Moreover, the transcription factor peroxisome proliferator-activated receptor-γ (PPAR-γ) promotes glucose uptake through an increased insulin sensitivity of the muscle, and a lower level of circulating lipids through an up-regulated storage of triglycerides [30]. 

Glucagon plays an important role in maintaining glucose homeostasis by promoting the breakdown of glycogen to glucose (glycogenolysis) and gluconeogenesis, and inhibiting glycogenesis, thereby acting as a glucose-mobilizing hormone [31,32]. It is released from pancreatic α-cells, when blood glucose levels start to decrease [27]. Similar to insulin secretion, the release of glucagon is triggered by Ca^2+^ entry through VDCCs. During a hypoglycemic state, low levels of glucose are taken up by GLUT-1 into the cell membrane of α-cells, which subsequently induces glycolysis resulting in low levels of ATP [31,33], being followed by the closure of the K_ATP_ channels, and thus, reduced efflux of K^+^ (Figure 2). Consequently, VDCCs open, allowing an influx of Ca^2+^ which triggers the release of glycogen from the α-cells [31,33]. Glucagon binds to the glucagon receptor, leading to a sequence of events [34,35,36,37,38,39] that convert glycogen to glucose (Figure 2). In addition to the promotion of glycogenolysis, glucagon inhibits glycogenesis in the liver simultaneously [34]. 

## 3. Type 2 Diabetes Mellitus

T2DM usually occurs in adults, but is increasingly seen in children and adolescents [40]. In T2DM patients, the pancreas produces and releases insulin, but the cells become resistant so that the insulin is ineffective, a state that is referred to as insulin resistance [IR]. Thus, the provided insulin may be insufficient to compensate for IR over time, a state that is referred to as relative insulin deficiency (ID) [41]. Both IR and ID lead to high blood glucose levels. T2DM patients also exhibit an impaired regulation of glucagon secretion, which is reflected in high levels during fasting in response to an oral intake of glucose [31,42,43,44]. The underlying mechanisms of hyperglucagonemia are currently not clear, but it may result from the impaired suppressive effect of insulin on the α-cells, due to hypoinsulinemia and IR [31,45,46].

### 3.1. Pathogenesis of T2DM

T2DM is characterized by two fundamental defects: Impaired insulin action (IR) in skeletal muscle, liver, and impaired adipocyte and β-cell function (Figure 1). It is caused by a combination of genetic factors related to impaired insulin secretion and IR, by environmental factors, such as obesity, lack of exercise, and stress, as well as by aging, indicating that T2DM is a multifactorial disease [27,47]. Several mechanisms for T2DM pathogenesis have been proposed, as described below.

#### 3.1.1. Oxidative Stress and T2DM

Oxidative stress is defined as the excess formation and/or insufficient removal of highly reactive molecules, that is, the reactive oxygen species (ROS) and reactive nitrogen species (RNS) [48]. The imbalance between the generation of ROS or RNS and the activity of the antioxidant defenses causes oxidative stress [49,50]. Mitochondria are integral to normal cellular function as they are responsible for energy production in eukaryotes, calcium homeostasis and also play a key role in the regulation of apoptosis [51,52]. Thus, alterations in mitochondrial function are often associated with T2DM, thus reflecting the centrality of energy homeostasis in β-cell physiology [53]. Clinical and experimental studies have shown that oxidative stress, through free radical generation, plays a major role in the onset of diabetes [54,55,56]. In high-sugar diets, mitochondria have more substrate available to generate ATP, due to the increased supply of glucose [57], resulting in an overproduction of their natural byproduct, ROS [15]. The increased ROS levels damage the infrastructure of the cell and induce mitochondrial stress [57] (Figure 1). The elevated ROS levels may also induce mitochondrial fission, which has been reported to cause mitochondrial dysfunction and IR in the skeletal muscle [58,59]. Hyperglycemia may also stimulate oxidative stress by the generation of ROS during the process of glycation, the non-enzymatic process through which glucose forms covalent adducts with plasma proteins, forming glycation end-products [60].

#### 3.1.2. Insulin Resistance

The predisposing factor and best indicator for the development of T2DM in the future is IR [57] (Figure 1). IR is classified into three categories, which are impaired insulin response in target tissues, diminished insulin secretion by β-cells, and insulin antagonists in the plasma [61]. IR is associated with an impaired insulin-dependent GLUT-4 translocation to the plasma membrane, which primarily arises from multifactorial defects in the normal engagement of the canonical insulin signaling cascade [14,25,62,63]. 

Obesity has been proposed as an underlying cause for the development of IR [64]. Chronic overfeeding leads to the elevated ability of adipose tissue to store the excess nutrients as triglycerides (possibly due to impaired insulin action), resulting in increased concentrations of circulating FFAs and abnormal redistribution of lipids to other organs, including the liver and skeletal muscle [64]. Elevated FFAs and intracellular lipids are linked to the onset of peripheral and hepatic IR [65,66]. This may result from the inhibition of insulin signaling by the FFAs and intracellular lipids, leading to a reduced insulin-stimulated muscle glucose transport, possibly due to a decrease in the translocation of the GLUT-4 [65,66]. Increased amounts of adipose tissue and visceral fat in obesity lead to ectopic fat accumulation in the liver, muscle, and pancreas, and thus, IR ensues [67]. 

Aberrant hepatic insulin action is hypothesized to primarily drive IR, given that higher circulating insulin levels are necessary to adequately control the blood glucose levels [68]. In patients with T2DM and obesity, insulin fails to appropriately regulate hepatic metabolism, leading to excess production of glucose despite accelerated rates of lipid synthesis, a condition commonly referred to as selective hepatic IR [69]. Hepatic IR is generally represented by the impaired suppression of hepatic glucose production, which is associated with an elevated hepatic triglyceride content, a known characteristic of non-alcoholic fatty liver disease (NAFLD) [70,71]. Other abnormalities associated with hepatic insulin resistance that may cause dysregulation of the glucose metabolism include the progression of simple steatosis (NAFLD) to fibrosis, and non-alcoholic steatohepatitis (NASH) [70,71].

#### 3.1.3. Pancreatic β-Cell Dysfunction/Failure

Several mechanisms describing the pathogenesis of pancreatic β-cell dysfunction/failure have been reported. Hyperglycemia and high amounts of saturated fats in circulation from diets or lipolysis of body fat have been suggested to trigger β-cell dysfunction, as well as IR [72] (Figure 1). Chronic hyperglycemia and elevated FFAs lead to β-cell dysfunction through various mechanisms, including the generation of ROS, increased intracellular Ca^2+,^ mitochondrial uncoupling, alterations in metabolic pathways, and the activation of endoplasmic reticulum stress [72,73]. Chronic exposure of β-cells to FFA is associated with impaired glucose-stimulated insulin secretion, a down-regulation of insulin gene expression resulting in reduced insulin synthesis, and ultimately causes apoptosis of the β-cells [73]. Chronic hyperglycemia causes an increased metabolic demand towards the β-cells, which undergo compensatory insulin hypersecretion to maintain normoglycemia [74]. This may lead to increased β-cell mass and function [73], consequently, to β-cell exhaustion and failure resulting in the development of T2DM [24,75,76]. Compensatory β-cell mass expansion may also be stimulated by increased FFAs consumption through increased production of glucagon-like peptide 1 (GLP-1) and its receptors as observed in dogs on a high-fat diet [77]. In addition to chronic hyperglycemia and elevated FFAs, obesity is a major risk factor for T2DM as it desensitizes glucose recipient organs to the action of insulin (obesity-induced IR), leading to increased insulin demand resulting in β-cell dysfunction [72]. 

## 4. Antioxidants and T2DM

Free radicals generated during biological oxidation reactions are reactive and simultaneously start the chain reaction, which may lead to damage or even to the death of cells [78]. An antioxidant is a substrate that prevents the oxidation of a molecule by neutralizing a free radical through the donation of an electron or by transferring a hydrogen atom, and thus, reducing its damaging potential [79]. Antioxidants are classified as either primary/chain-breaking/radical-trapping (slow-down/block autoxidation by competing with the propagation reactions) or secondary/preventive (interfere with the initiation process) [80,81,82]. The primary antioxidants (e.g., phenolic compounds, such as caffeic acid and tocopherol) rapidly react with peroxyl radicals preventing their reaction with oxidizable substrates and consequently the propagation of the autoxidation chain [80,82]. Secondary antioxidants (e.g., polyphenols, including flavonoids, such as quercetin [83]) may prevent the occurrence of Fenton-type chemistry by blocking redox-active metal ions in an oxidized form (e.g., Fe^3+^) through metal chelation [82,84]. 

### Endogenous and Exogenous Antioxidants in Humans 

The antioxidant defense grid in living systems consists of antioxidant molecules that act at different levels and are classified as the first-line, second-line, third-line, and fourth-line [79,85,86]. These are radical suppression or prevention, radical scavenging, radical-induced damage repair, and adaptation (utilization of the signals required for free radical production by reacting to prevent the formation or reaction of the radicals) [85], respectively. First-line antioxidants include superoxide dismutase (SOD), catalase (CAT), glutathione peroxidase (GPx), and glutathione reductase (GR) [85,87]. Second-line includes hydrophilic antioxidants (such as ascorbic acid, uric acid, phenolics), and glutathione and lipophilic antioxidants (such as vitamin E and ubiquinol) [85,88]. Third-line includes proteolytic enzymes, lipases, DNA repair enzymes, and transferases [79,85,86].

The human endogenous antioxidant defense against free radicals and oxidative stress includes enzymatic antioxidants, such as SOD, which catalyzes the dismutation of superoxide (O_2_^−^) radical into either ordinary molecular oxygen (O_2_) or H_2_O_2_, as well as CAT and GPx, which both remove H_2_O_2_ [87,89], and non-enzymatic antioxidants, such as lipoic acid, glutathione, L-arginine, and coenzyme Q10 [87,90]. However, these endogenous antioxidants may not be sufficient in some cases, such as chronic exposure to free radicals, due to smoking and consumption of high nutrient diets. Thus, exogenous (dietary) antioxidant consumption may help in the prevention of diseases associated with oxidative stress [91]. Sources of dietary antioxidants include herbs, spices, medicinal plants [92], fruits, and vegetables [93]. Due to the presence of antidiabetic phytochemicals [94], medicinal plants are used as antidiabetic remedies worldwide [95]. Fruits and vegetables are also considered protective, due to various phytochemicals that are mainly responsible for the plant’s color, smell, flavor, and bitterness [96], such as polyphenols, alkaloids, and saponins. Phytochemicals are defined as bioactive plant chemicals that may provide desirable health benefits that lower the risk of developing major chronic diseases [97], including T2DM. This may be achieved by reducing cholesterol absorption, by directly lowering fasting blood glucose levels, e.g., by inhibiting cortisol [98,99], and by stimulating the immune system under different conditions [100]. The antioxidant activity of foods correlates with the presence of phytochemicals [100]. Thus, in addition to the enzymatic antioxidants (CAT, SOD, GPx) in humans that scavenge free radicals, some phytochemicals also act as complementary antioxidants, due to their electrophilicity, ability to promote the gene expression of antioxidant enzymes, and to positively modulate the actions of antioxidant enzymes [101]. 

## 5. *Solanum anguivi* Lam. Fruit’s Antidiabetic Properties and Potential Mechanisms of Action

Various researchers [92,102,103,104,105] have reported the presence of phytochemicals in SALF, which include phenolics, flavonoids, saponins, alkaloids, coumarins, and vitamin C. The phenolics in SALF include gallic acid, chlorogenic acid, caffeic acid [92], phenolic acids [106], and tannins [102], as well as rutin and quercetin as representatives of the flavonoids [92]. Triterpenoid saponins [102] and steroidal saponins or glycosides, such as anguiviosides A to C [107], III, XI, XV, and XVI [108] have also been reported to be present in SALF. In addition, the steroidal glycoalkaloids solamargine, anguivine, and isoanguivine have been described to be present in the SALF [109,110]. There is controversy about whether *Solanum indicum* Linn. (*S. indicum*) is the same as *Solanum anguivi* Lam. (*S. anguvi*). *S. indicum* has been reported as a synonym for *Solanum anguivi* by some authors [111,112,113,114], while others have described them as different species [106,115,116]. Controversy also exists regarding the safety of *S. indicum* L. fruit (SILF). Several authors have reported that SILF is safe, and thus, may be consumed as a vegetable [9,113,114,117,118,119], while one author has reported that it is a poisonous berry [115]. Similar to SALF, SILF has been reported to contain steroidal saponins/glycosides (isoanguivine, protodioscin, solasonine, solamargine, and indiosides A–E), terpenoids, vitamin C, phenolics (gallic acid, catechin, chlorogenic acid, caffeic acid, epicatechin), flavonoids (rutin, quercetin, isoquercitrin), glycoalkaloids (solamargine, solasonine) and coumarins [120,121,122]. The similarities between the phytochemicals present in SALF and SILF may, therefore, indicate similarities in their antidiabetic properties.

**Figure 3:**
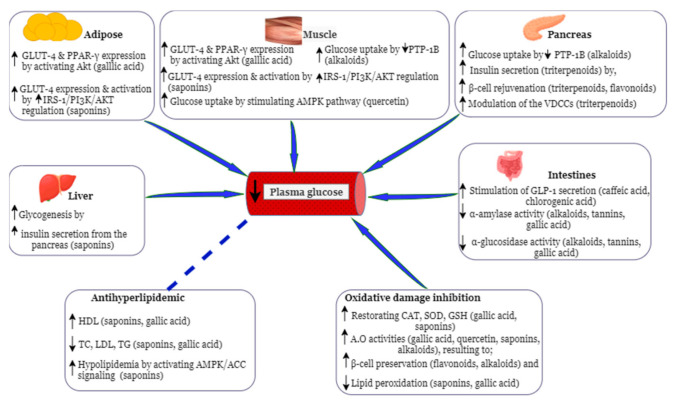
Potential mechanisms of action by *Solanum anguivi* Lam. Fruit phytochemicals in their antidiabetic effects. PPAR = peroxisome proliferator-activated receptors, GLUT = glucose transporter, IRS = insulin receptor substrate, AMPK = adenosine monophosphate-activated kinase, ACC = acetyl-CoA carboxylase, PI3-K = phosphatidylinositol-3-kinase, VDCCs = voltage-dependent calcium channels, GLP = glucagon-like peptide, PTP-1B = protein tyrosine phosphatase-1B, CAT = catalase, SOD = superoxide dismutase, GSH = glutathione, A.O = antioxidant, upward black arrows = increased, downward black arrows = inhibition of, blue arrows = mechanisms of action resulting in reduced plasma glucose levels, stripped blue line = relationship between hypoglycemia and hypolipidemia. The figure was drawn via https://app.diagrams.net/, and the pancreas, liver, muscle, and intestine pictures were obtained from www.freepik.com.

Previous studies have shown that SALF extracts possess antioxidant abilities in vitro, such as radical scavenging capacity [92,105], reducing properties [Fe^3+^ to Fe^2+^], and iron-chelating abilities [92,123]. SALF extracts have also been reported to inhibit lipid peroxidation [92], which may be due to the presence of saponins as they have been reported to inhibit lipid peroxidation in diabetic rats through the restoration of SOD and CAT [104]. Blood-glucose-lowering effects have also been exhibited in diabetic rats having been administered SALF extracts [5,6]. The antidiabetic properties (antioxidant activities, inhibition of oxidative stress, and blood-glucose-lowering effect) of SALF may be attributed to the presence of various phytochemicals in SALF. However, only one class of phytochemicals present in SALF, that is, saponin, has been studied for its antidiabetic effects [5,104,124]. Since the antidiabetic properties of the other SALF phytochemicals (phenolics, flavonoids, and alkaloids) have not been documented, we discuss their potential antidiabetic effects and underlying mechanisms of action (summarized in Figure 3) in the context of other medicinal plants with similar phytochemical patterns.

### 5.1. Saponins

The total saponin content of SALF has been stated as 1.3 mg/100 g dry weight (DW) and the triterpenoid content as 0.3 mg/100 g DW [102]; however, no reference standard (n.r.s) was used for both analyses. Other authors have also reported the presence of saponins in SALF, however, not the total saponin content [103,125]. Saponins extracted from SALF have been stated to exhibit antioxidative properties in vitro, such as scavenging radicals, reducing [Fe^3+^ to Fe^2+^], and iron-chelating abilities [104]. Furthermore, SALF saponins have been reported to exhibit antidiabetic properties in diabetic rats, including the reduction of blood glucose levels [5], and the inhibition of oxidative stress [5,104,124], which may both be due to their ability to restore the endogenous antioxidant levels (i.e., SOD and CAT levels) [5,104,124], as well as their antioxidative activities [104]. The antidiabetic effect of SALF saponins may also be referred to as their antihyperlipidemic properties and their ability to cause weight loss in diabetic rats [5]. SALF saponins have also been reported to restore the plasma lipid profile in these rats, being reflected in lower levels of total cholesterol (TC), triglycerides (TG), and low-density lipoprotein (LDL), and increased levels of high-density lipoprotein (HDL) [5]. Relative ID and IR may negatively affect the lipid profile as insulin plays a critical role in lipid homeostasis [126]. In T2DM patients, elevated plasma levels of TG and lipoprotein lipase (LPL), as well as decreased HDL levels, have been found, the latter being associated with defective LPL catabolism of TG-rich lipoproteins [127]. Similar to diabetic rats, being exposed to SALF saponins [5], an improvement of glucose homeostasis in the absence of weight gain has been suggested to result in lowered TG and increased HDL levels [127]. In contrast, other authors have suggested that hyperglycemia may not cause dyslipidemia, but rather abnormalities in insulin action, and hence, a hypoglycemic effect may not improve the lipid profile per se [128,129].

Another possible mechanism through which saponins from SALF may result in hypoglycemia may be through the regeneration of islets of Langerhans as suggested for *Solanum nigrum* (*S. nigrum*) after being administered to diabetic rats [130]. Triterpenoid saponins in SALF may also cause hypoglycemic and hypolipidemic effects by activating GLUT4 through improved IRS-1/PI3K/AKT regulation, and activated adenosine monophosphate-activated kinase/acetyl-CoA carboxylase (AMPK/ACC) signaling, respectively, as shown in diabetic mice by *Stauntonia chinensis* triterpenoids [131]. Additionally, SALF triterpenoid saponins may lower plasma glucose levels by improving insulin secretion as a result of the improved modulation of VDCCs, and thus, increasing glycogenesis, and β-cell rejuvenation, as reported for *Primula denticulate* [132] and *Momordica cymbalaria* Fenzl [133] triterpenoids. 

### 5.2. Phenolics and Flavonoids

The total phenolic content (TPC) for SALF has been reported as 17.1 mg gallic acid equivalent (GAE)/g dry weight (DW) [92], 1.52 mg/100 mg DW (1.52%) (n.r.s) [102], and from unripe to very ripe stage, 9.6 to 5.5 mg/g DW (n.r.s) [103] and 11.6 to 4.5 mg/g GAE DW [134], respectively. The total flavonoid content (TFC) of SALF has been documented as 9.5 mg QE/g DW [92], 0.5 mg/100 mg DW (0.5%) (n.r.s) [102] and 141.3 to 455.0 mg QE/100 g DW from unripe to very ripe stage, respectively [134]. Elekofehinti et al. [92] described the contents for the SALF phenolic compounds gallic acid, chlorogenic acid, caffeic acid, rutin, and quercetin as 17.5, 21.9, 16.6, 14.7, and 7.4 mg/g, respectively. Stommel et al. [106] reported the contents (µmol/100 g DW) of SALF chlorogenic acid isomers (1117–6232), isochlorogenic acid isomers (70–226), hydroxycinnamic acid amide conjugates (14–286), caffeic acid derivatives (45–155), and acetylated chlorogenic acid isomers (316–1148). The tannin content of SALF has been documented as 0.17 mg/100 mg (n.r.s) [102] and 0.19 to 0.09 mg tannic acid equivalent/100 g DW, from unripe to ripe stage, respectively [134]. Extracts from SILF and *Solanum melongena* (*S. melongena*) have been stated to exhibit antidiabetic properties through inhibiting α-amylase and α-glucosidase enzymes, which was attributed to the present phenolics [120,135]. The TPC and TFC of SILF were reported as 3.8 mg GAE/g DW and 1.7 mg quercetin equivalent (QE)/g DW, respectively [120], while the TPC and TFC for *S. melongena* were only reported for the skin and pulp separately. Glucose is a product from the hydrolysis of starch [136], which is catalyzed by the enzymes α-amylase found in saliva and pancreatic juices, and α-glucosidase found in the epithelium of the small intestine [137]. Hence, α-amylase and α-glucosidase inhibitors slow the digestion of starch in the small intestine, which decreases the amount of glucose entering the bloodstream leading to an improved insulin response [138]. Previous studies reported the hypoglycemic effects of SILF [139] and *S. melongena* [140], and these may have been mediated through inhibiting the α-amylase and α-glucosidase, due to the presence of phenolic compounds. The TPC in SALF (mg GAE/g DW) is substantially higher than in SILF [120], suggesting inhibiting properties of SALF regarding α-amylase and α-glucosidase activity, which may be mediated by tannins as similar effects have been reported for tannin-containing *Terminalia chebula* Retz [141].

Phenolics present in SILF, *S. nigrum*, *S. melongena,* have been reported to possess antioxidant effects [120,135,140,142], which may also be true for SALF phenolics. Thus, in addition to saponins, the antioxidative effect of SALF extracts may be induced by the synergistic action of saponins and phenolics. Polyphenols, such as gallic acid, may also be responsible for SALF’s antidiabetic effects. They may also be mediated through both a reduction of plasma glucose levels and oxidative stress damage, by restoring antioxidant enzymes, inhibiting α-amylase and α-glucosidase, as well as by maintaining a healthy lipid profile as already shown in diabetic rats for *Hibiscus sabdariffa* gallic acid [143]. Furthermore, SALF gallic acid may increase the expression of GLUT-4 and insulin sensitivity proteins, such as PPAR-γ, through the activation of AKT as demonstrated for *Emblica officinalis* derived gallic acid in diabetic mice [144], consequently leading to increased cellular glucose uptake. Recent studies [145,146] have shown that polyphenols increase GLP-1, suggesting them to be used together with GLP-1 agonists for the treatment of T2DM [147,148]. GLP-1, an incretin hormone produced from proglucagon in the intestine and brain [31,147,149], stimulates insulin release, the proliferation and neogenesis of pancreatic β-cells, and inhibits glucagon release, food intake, and gastric emptying [149,150,151]. Potentially SALF may stimulate GLP-1 secretion through its polyphenols, such as caffeic and chlorogenic acid [145,152,153]. 

Flavonoids from SALF may also possess antidiabetic effects. The hypoglycemic effect and the regeneration of islets of Langerhans in diabetic rats administered with extracts of *S. nigrum* were referred to flavonoids in the extract [130], whose TFC has been reported as 3.61 mg QE/g DW [154]. This could also apply to SALF flavonoids, which potentially exhibit antioxidant properties, protect against oxidative damage and restore pancreatic cells, which result in decreased levels of glucose in the blood. SALF quercetin may increase glucose uptake in skeletal muscles by stimulating the insulin-independent AMPK pathway, which has been demonstrated by quercetin-containing *Vaccinium vitis-idaea* in vitro [155].

### 5.3. Alkaloids

Alkaloids have also been reported to possess antidiabetic properties [156]. Although SALF has been shown to possess alkaloids (0.05 mg/100 mg DW or 0.05% [102]), there is very limited literature on the antidiabetic effects of alkaloids from *Solanum* fruits. However, SALF alkaloids may lower blood glucose levels as shown for *Aerva lanata* alkaloids in diabetic rats [157]. This may be through inhibiting α-amylase and α-glucosidase activities as suggested for *S. melongena* alkaloids [158]. SALF alkaloids may also lower blood glucose levels by inducing glucose uptake through inhibition of protein tyrosine phosphatase-1B (PTP-1B) (a major negative regulator for insulin receptor signaling [159]) as demonstrated in C2C12 skeletal muscle cells by alkaloids from *Veratrum nigrum* [160] and *Catharanthus roseus* [161], and in β-TC6 pancreatic cells by alkaloids from *Catharanthus roseus* [161]. Additionally, SALF alkaloids may also alleviate H_2_O_2_-induced oxidative damage in β-cells as shown by alkaloids from *Catharanthus roseus* in diabetic rats, due to its radical scavenging capacity [161].

## 6. Conclusions

Some studies have documented antidiabetic effects of SALF. For one group of phytochemicals present in SALF, the saponins, the antidiabetic effect, and the underlying mechanism have been documented. As SALF also contains other phytochemicals, such as phenolics, flavonoids, and alkaloids, its antidiabetic effect may also refer to these compounds, which have been shown to decrease blood glucose levels through, e.g., an up-regulation of GLUT-4 and PPARγ, restoration of enzymatic antioxidants and β-cell regeneration in other settings. However, to unravel the precise underlying mechanisms of the potential antidiabetic effects of SALF, further studies are essentially needed. They would provide information on whether the SALF antidiabetic properties may be due to a potential synergistic action of saponins, and other phytochemicals present, or refer to the saponin fraction only. Consequently, the results may also provide valuable information on the potential use of SALF in T2DM management.
